# Supplementary figures and images for: Beyond peak wavelength: spectral bandwidth of blue and red-blue laser diodes modulates photosynthesis, canopy architecture, chlorophyll maintenance, and whole-plant growth
Source: Front Plant Sci. 2026 Jun 10;17:1817114. doi: 10.3389/fpls.2026.1817114 (PMC13291052; doi:10.3389/fpls.2026.1817114)

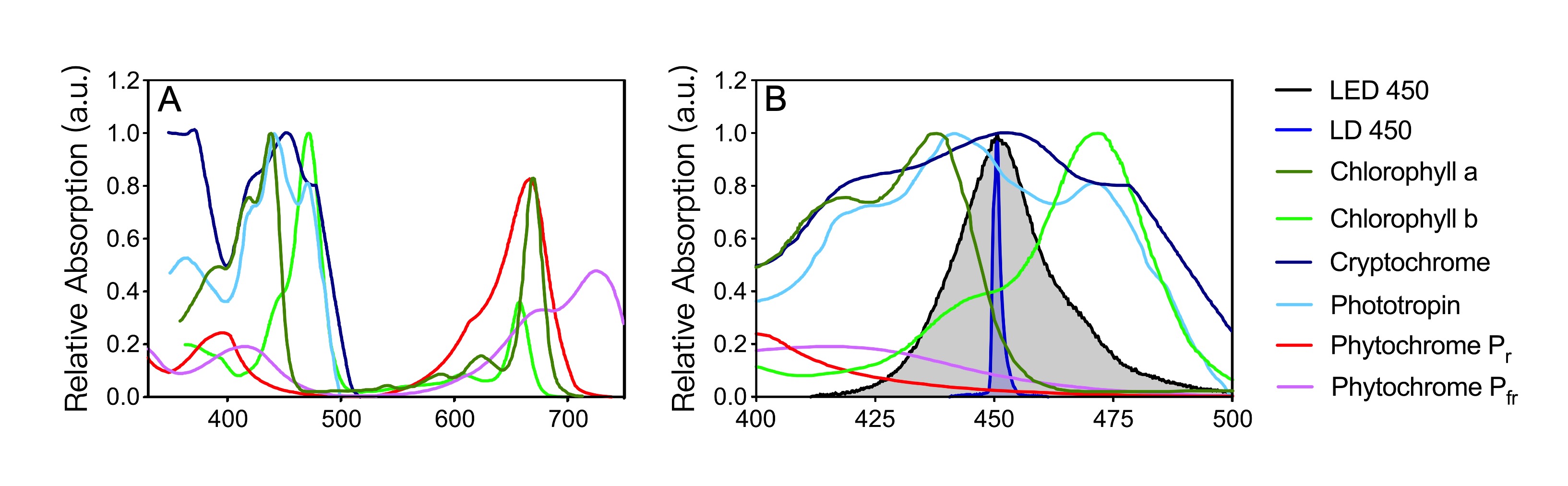

Supplement: Supplementary Figure 1 — Blue light source spectra and the absorption spectra of key plant pigments and photoreceptors. (A) Normalized absorption spectra for major photosynthetic pigments (Chlorophyll a, Chlorophyll b) and key photoreceptors (Cryptochrome, Phototropin, Phytochrome Pr, and Phytochrome Pfr). (B) Spectral distributions of the LED 450 and LD 450 light sources, overlaid with the absorption spectra of pigments and photoreceptors active in the 400–500 nm range. [file Image1.jpg]

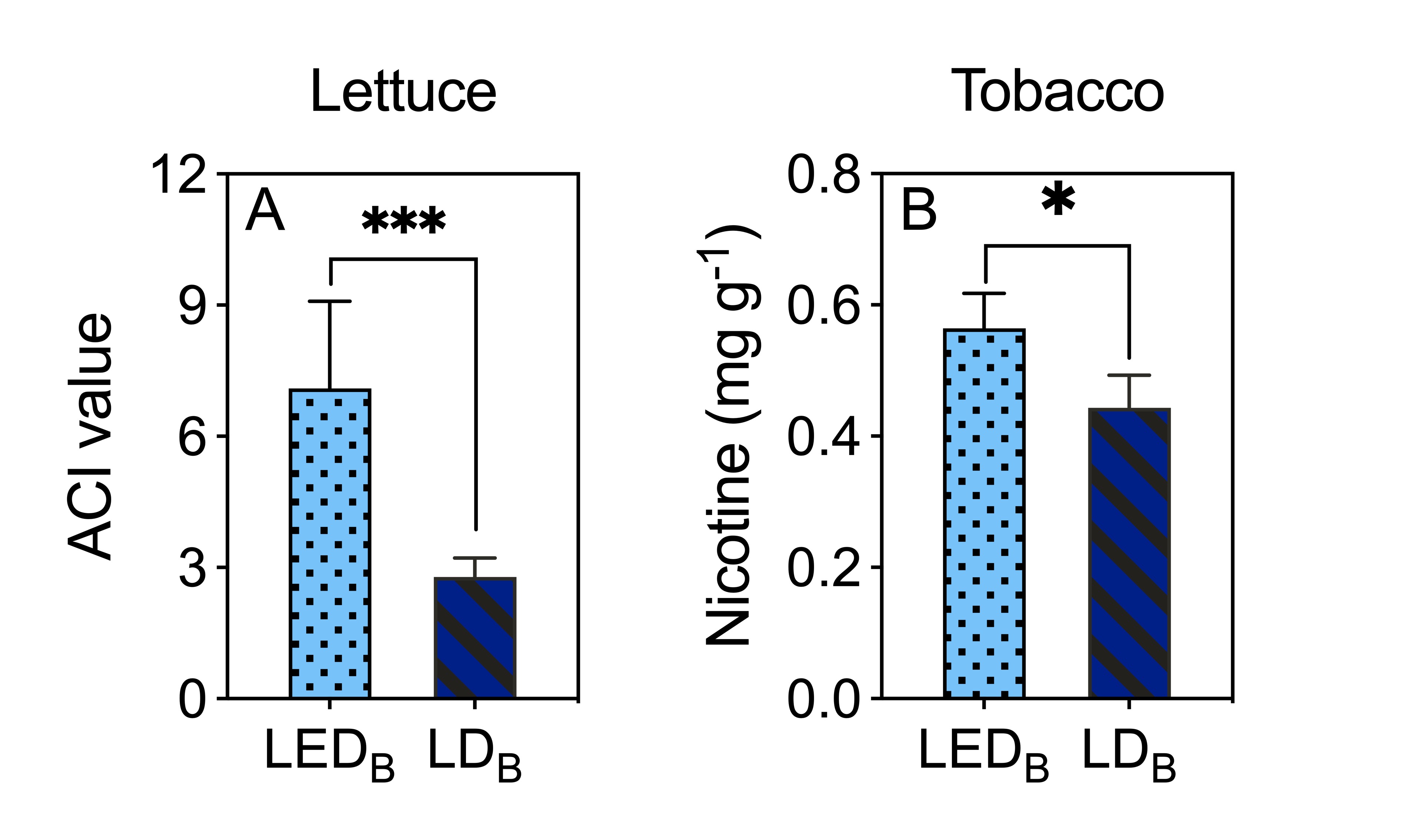

Supplement: Supplementary Figure 2 — Secondary metabolite accumulation in tobacco and lettuce under monochromatic blue light. Treatments consisted of a monochromatic blue LED (LEDB) or a blue LD (LDB), both with an identical peak wavelength at 450 nm, applied at a PPFD of 150 μmol·m-²·s-¹. (A) Anthocyanin accumulation (ACI value; leaf area based) in lettuce plants after 12 days of continuous irradiation. (B) Nicotine content of tobacco leaves after 8 hours of irradiation. Data are presented as the mean ± SE (n = 4). Asterisks indicate significant differences between treatments (* P < 0.05, *** P < 0.001; t test). [file Image2.jpg]

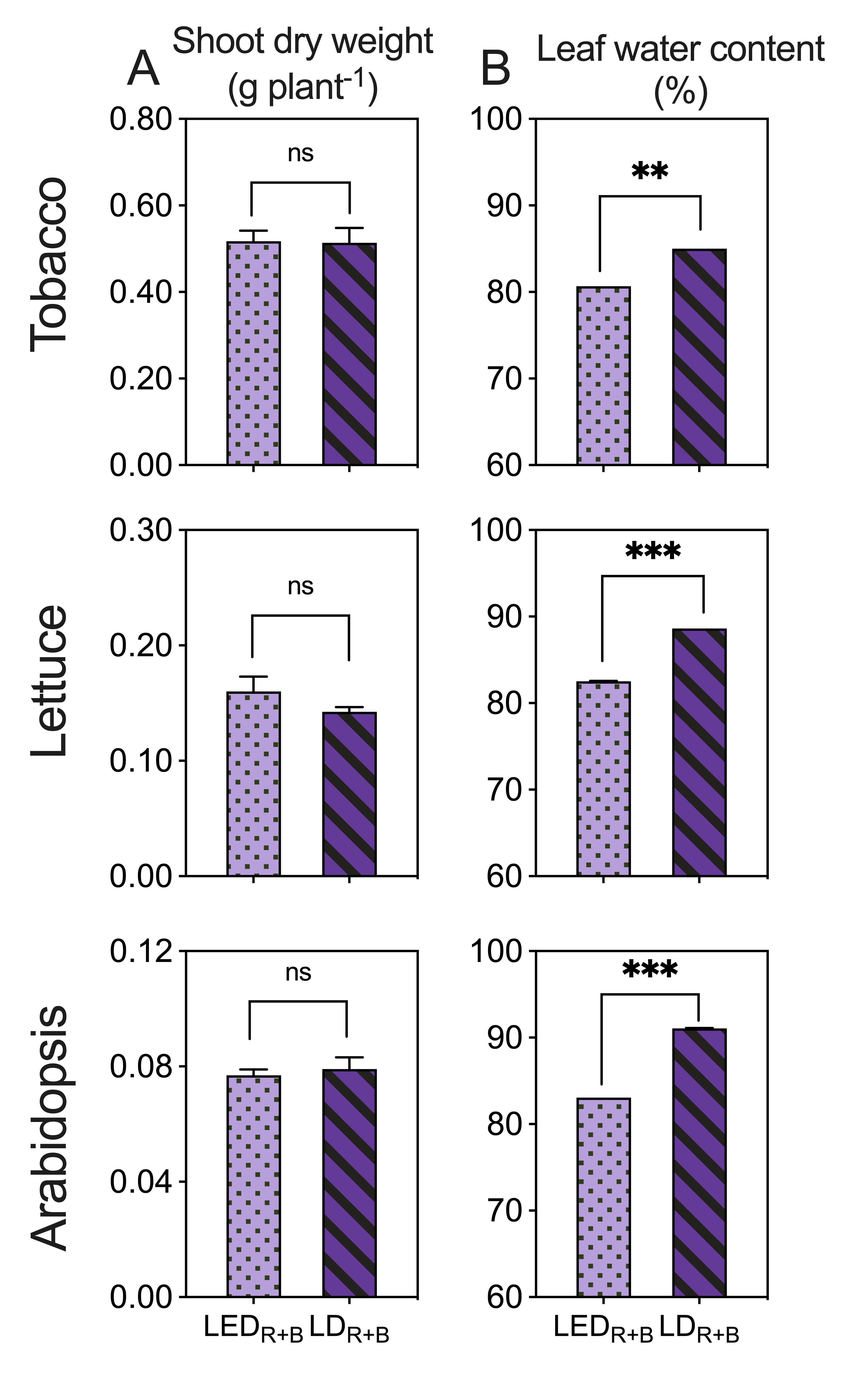

Supplement: Supplementary Figure 3 — Shoot dry weight (A) and leaf water content (B) of tobacco, lettuce, and Arabidopsis under continuous combined red and blue light. Plants were grown for 15 days under either a LED (LEDR+B) or a LD (LDR+B) at of PPFD of 150 μmol·m-²·s-¹. Data are presented as the mean ± SE (n=4). Asterisks indicate significant differences between treatments (** P < 0.01, *** P < 0.001; t test). ns indicates no significant difference. [file Image3.jpeg]
