# Supplementary material for: Beyond peak wavelength: spectral bandwidth of blue and red-blue laser diodes modulates photosynthesis, canopy architecture, chlorophyll maintenance, and whole-plant growth
Source: Front Plant Sci. 2026 Jun 10;17:1817114. doi: 10.3389/fpls.2026.1817114 (PMC13291052; doi:10.3389/fpls.2026.1817114)
Supplement: Supplementary Table 1 — Spectral characteristics of LED 450 and LD 450. [file Table1.docx]

Supplementary Table 1. Spectral characteristics of LED 450 and LD 450

| Treatments | FWHM^1^ | PPFD^2^ | YPFD^3^ | PPE^4^ |
| --- | --- | --- | --- | --- |
| LED 450 | 20.1 | 150 | 110.7 | 0.4861 |
| LD 450 | 1.6 | 150 | 112.5 | 0.4906 |

^1^FWHM: Full width at half maximum of light spectrum (nm).

^2^PPFD: Photosynthetic photon flux density (μmol m^-2^ s^-1^).

^3^YPFD: Yield photon flux density (μmol m^-2^ s^-1^), calculated as the product of the incident flux density of spectral photon distribution (SPD) and relative quantum efficiency, based on McCree (1972) and Sager et al. (1988).

^4^PPE: Phytochrome photoequilibria, representing the estimated P_r_/P_total_ calculated following Sager et al. (1988).
